# Re-experiencing traumatic events in PTSD: new avenues in research on intrusive memories and flashbacks

**DOI:** 10.3402/ejpt.v6.27180

**Published:** 2015-05-19

**Authors:** Chris R. Brewin

**Affiliations:** Clinical Educational & Health Psychology, University College London, London, United Kingdom

**Keywords:** Posttraumatic stress disorder, memory, flashbacks

## Abstract

Posttraumatic flashbacks, consisting of the intrusive re-experiencing of traumatic experiences in the present, have been more clearly defined for the first time in DSM-5 and have been identified as a unique symptom of posttraumatic stress disorder in the proposed ICD-11 diagnostic criteria. Relatively little research into flashbacks has been conducted, however, and new research efforts are required to understand the cognitive and biological basis of this important symptom. In addition, there is considerable scope for research into how flashbacks should be assessed and into flashbacks occurring in different contexts, such as psychosis or intensive care.

When the posttraumatic stress disorder (PTSD) diagnosis was first introduced into the DSM-III in 1980, there were several symptoms that appeared to be distinctive, including frequent intrusive recollections of the traumatic event and acting or feeling as though it were happening again (dissociative flashbacks). It is now evident that frequent intrusive recollections of stressful and unpleasant events, many of them traumatic, are found in most forms of psychopathology (Brewin, Gregory, Lipton, & Burgess, 2010). Instead, evidence is gradually accumulating that it is the reliving in the present that distinguishes intrusive memories in PTSD from those in other disorders (Brewin, 2014), and this insight has been incorporated in proposed revisions to the PTSD diagnosis for ICD-11 (Maercker et al., 2013). Despite these developments, understanding of traumatic flashbacks remains limited, offering opportunities for new research.

Pierre Janet, one of the first traumatologists, wrote extensively about how memories of traumatic experiences are dissociated or split off from normal consciousness, resulting in powerful and uncontrollable re-enactments of the events (Van der Kolk & Van der Hart, 1989). Frequently remarked characteristics of this traumatic re-experiencing in PTSD are its involuntary and uncontrollable nature, the strong sensory impressions, and the sense of “nowness” or of the event occurring in the present (Brewin, Dalgleish, & Joseph, 1996; Ehlers, Hackmann, & Michael, 2004), phenomena that can be equally observed in children with posttraumatic conditions (McKinnon, Nixon, & Brewer, 2008; Meiser-Stedman, Dalgleish, Smith, Yule, & Glucksman, 2007). The sense of nowness also distinguishes involuntary memories in PTSD from the involuntary memories reported by depressed patients or individuals who were exposed to trauma without developing PTSD (Birrer, Michael, & Munsch, 2007; Reynolds & Brewin, 1998).

Re-experiencing trauma memories in the present is predictive of the course of the disorder over and above the effects of initial symptom levels (Kleim, Ehlers, & Glucksman, 2007; Michael, Ehlers, Halligan, & Clark, 2005) and decreases with successful treatment (Hackmann, Ehlers, Speckens, & Clark, 2004; Speckens, Ehlers, Hackmann, & Clark, 2006). Moreover, specifically addressing flashbacks in therapy appears to contribute to better outcomes (Nijdam, Baas, Olff, & Gersons, 2013). Although this form of memory may represent a normal short-term response to trauma exposure, and may occasionally be encountered in other disorders (for example, where there is a trauma history), empirical studies confirm that continuing to experience flashbacks appears to be a specific indicator of PTSD (Bryant, O'Donnell, Creamer, McFarlane, & Silove, 2011).

## Defining flashbacks

Lack of any formal definition of flashbacks or dissociative re-experiencing resulted in uncertainty about whether the term should be reserved for extreme episodes in which individuals completely lose contact with their surroundings for periods of minutes or more, or whether they should include all intrusive memories that are accompanied by a sense of reliving the event in the present, even if only fleeting. Both DSM-5 and the proposed ICD-11 PTSD criteria have now opted for the more inclusive definition in which flashbacks are seen as existing on a continuum between these two extremes. This is an important step forward which will enable researchers to communicate their findings more effectively.

## Flashbacks or ordinary autobiographical memories?

Although some researchers regard flashbacks as no different in principle from other autobiographical memories (Rubin, Berntsen, & Bohni, 2008), clinical observations are supported by studies that explicitly contrast flashback or reliving experiences with ordinary autobiographical memories for the same event. In an initial study, Hellawell and Brewin had PTSD patients write a detailed narrative of their traumatic event. They then defined a flashback for them as follows: “A type of memory that you experience as markedly different from those memories of the event that you can retrieve at will. The difference might be a marked sense of a reliving of the traumatic experience(s). Some report complete reliving, whereas others report more momentary or partial reliving of perhaps just one aspect of the original experience. For some, flashback memories take them by surprise or swamp their mind. Finally, some report a sense of time-distortion and, for example, react to the flashback memory as though it was an event that was happening in the present.” Patients in the study then went back over their narrative armed with this definition and distinguished individual words and phrases that had been accompanied by flashbacks from those that were not (Hellawell & Brewin, 2002).

Although this may seem like a difficult task to perform on a narrative that might take 30–40 min to write, PTSD patients did not appear to find it difficult, and the validity of their categorisations using this method has been demonstrated on many different measures. For example, while writing sections that would later be classified as flashbacks, patients were observed to display significantly more involuntary motor responses. Patients interrupted in the middle of these sections to carry out a separate visuospatial task also performed significantly worse on the task than patients interrupted during ordinary memory sections (Hellawell & Brewin, 2002). Subsequent studies have shown that flashback sections are rated as more negative and arousing (Brewin, Huntley, & Whalley, 2012); are accompanied by increases in heart rate (Chou, La Marca, Steptoe, & Brewin, Manuscript submitted for publication); and also contain more sensory words, mentions of death, and the core PTSD emotions of fear, helplessness, and horror (Hellawell & Brewin, 2004). Like the real-life situations PTSD patients encounter, the same words and phrases tend to elicit flashbacks repeatedly, but not invariably—flashback elicitation is a probabilistic rather than a predictable process (Brewin et al., 2012).

## Development of a neurobiological theory of flashbacks

It is likely that the scientific investigation of flashbacks will benefit from theories that, like the revised dual representation model (DRT: Brewin et al., 2010), relate clinical observations to established brain pathways and make specific predictions about the neural basis of this type of memory. The utility of such models is likely to depend on the success with which they can integrate evidence from conditioning, autobiographical memory, and imaging studies with clinical observations.

There is now a considerable quantity of evidence supporting the idea that flashbacks depend on the involvement of an involuntary perceptual memory system that is distinct from ordinary episodic memory (Brewin, 2014). Normal episodic memory is thought to depend on focussing conscious attention onto objects and scenes such that, by virtue of sharing the same location in space, individual features are bound together to create a stable, contextualised representation that can be retrieved or inhibited at will (Treisman & Gelade, 1980). During traumatic events, however, attention tends to be restricted and focused on the main source of danger, so that sensory elements from the wider scene encoded by the perceptual memory system will be less effectively bound together, producing fragmented and poorly contextualised memories that are difficult to control. Laboratory research has shown that such unattended patterns or events, providing they are sufficiently novel, produce long-lasting memory traces. The existence of these traces can be detected, for example, through facilitation or negative priming effects on re-presentation of the stimuli, even though a memory of the original pattern cannot be deliberately retrieved (DeSchepper & Treisman, 1996).

### Encoding under acute stress

According to the revised DRT, flashbacks depend on a stress-related excess of activity in the dorsal visual stream, which is specialised for creating images of the environment from a first-person perspective that can be used, among other things, to direct immediate motor responses to threat. High levels of stress also lead to a corresponding reduction of activity in the ventral visual stream and medial temporal lobe, where the elements of objects and scenes are normally bound together and encoded in an abstract form that enables them to be identified, manipulated, and related to past experience. The result is poorly contextualised, fragmented images and scenes that when triggered by trauma reminders are experienced as flashbacks. Consistent with this, acute stress has been found to impair performance on spatial learning tasks dependent on medial temporal lobe processing, both in healthy individuals (Meyer, Smeets, Giesbrecht, Quaedflieg, & Merckelbach, 2013) and PTSD patients (Smith, Burgess, Brewin, & King, 2015).

It is interesting to speculate about the intensity of stress that would lead to an opposite pattern of activity in the visual streams (and possibly in other sensory processing streams) sufficient to produce flashbacks. A candidate is panic attacks, which are a common occurrence during the traumatic event in individuals who subsequently develop PTSD (Joscelyne, McLean, Drobny, & Bryant, 2012). In turn, there is evidence that flashbacks are involved in the maintenance of orthostatic panic, that is, panic upon standing, which is a key complaint among traumatised Cambodian refugees (Hinton et al., 2010).

### Neuroimaging studies

Unlike standard models of autobiographical memory, which predict that powerful and vivid memories should be associated with additional activity in medial temporal lobe structures such as the hippocampus, dual representation theory predicts that flashbacks should be associated with increased activity in motor areas, insula, and amygdala, but reduced medial temporal lobe activity. In one study, subjective flashback intensity was correlated with regional cerebral blood flow in 11 PTSD patients (Osuch et al., 2001). They observed flashback-related increases in left inferior frontal cortex and bilateral insula, and flashback-related decreases in right medial temporal and right ventral stream structures such as fusiform cortex. Echoing these results, a study using Hellawell and Brewin's (2002) technique for distinguishing flashbacks and ordinary episodic memories found that flashbacks were associated with increased activation in sensory and motor areas including the insula, precentral gyrus, supplementary motor area, and mid-occipital cortex, but with decreased activation in a medial temporal area, the parahippocampal gyrus (Whalley et al., 2013). Together with evidence that patients reporting more flashbacks have reduced brain volume in areas of the visual ventral stream (Kroes, Whalley, Rugg, & Brewin, 2011), these studies provide tantalising suggestions that flashbacks are supported by patterns of neural activity that distinguish them from ordinary autobiographical memories.

## Future research

### Assessment of flashbacks

An unanswered question is whether flashbacks are currently assessed in the most appropriate way. As they are typically a response to an internal or external cue, questionnaire items about their frequency in the past week or month may not be the most accurate method of assessing them. Apart from the fact that retrospective judgments are often not very reliable (Priebe et al., 2013), high levels of avoidance may temporarily be suppressing these symptoms. One possibility is to ask a more specific question about whether individuals would experience flashbacks if they allowed themselves to fully remember the event or confront reminders of it. We also know little about what patients understand by typical questions such as “Have you ever suddenly acted or felt as if the event(s) was happening again?”, and whether different forms of wording would elicit more accurate answers.

### Underlying mechanisms

In addition to investigating the cognitive and neurobiological basis of flashbacks in PTSD using theories such as the DRT, it is also likely that flashbacks occur in other patient groups and could add to our understanding of the underlying mechanisms. For example, pain flashbacks have been described in which it is somatic rather than visual sensations that are repeatedly re-experienced as though they were happening in the present (Salomons, Osterman, Gagliese, & Katz, 2004; Whalley, Farmer, & Brewin, 2007). Frightening delusions and hallucinations, such as occur in psychosis or to intensive care patients, can also give rise to traumatic re-experiencing (Berry, Ford, Jellicoe-Jones, & Haddock, 2013; Gracie, Hardy, Brewin, & Fornells-Ambrojo, Manuscript submitted for publication; Wade et al., 2014). Although hallucinations do not meet current DSM-5 criteria for a traumatic event, the fact that the person has had a terrifying experience and developed characteristic symptoms thereafter would permit a diagnosis of PTSD within ICD-10 or ICD-11.

## Towards ICD-11

PTSD, compared to other disorders, is excessively complex to diagnose, and this very complexity is likely to impede clinical and scientific advances. It makes sense both to simplify the diagnosis as far as is possible, and to focus on the mechanisms responsible for key symptoms that maintain the disorder and are treatment targets in their own right. Along with this should go more consistent terminology, so that “involuntary autobiographical memories” (an everyday memory phenomenon), “intrusive memories” (involuntary memories with repeated and usually distressing content, generally associated with psychological disorders), and “flashbacks” (involuntary memories involving re-experiencing distressing events in the present, thought to be specific to PTSD) are not used interchangeably (Kvavilashvili, 2014).

Under the ICD-11 proposals for the PTSD diagnosis just six symptoms have been identified (one of them being flashbacks) that are thought to be the most reliable indicators discriminating PTSD from other disorders (Brewin, Lanius, Novac, Schnyder, & Galea, 2009; Maercker et al., 2013). Preliminary evidence suggests that if the ICD-11 proposals were implemented, PTSD prevalence would remain largely unchanged (Morina, Van Emmerik, Andrews, & Brewin, 2014; Stein et al., 2014; Van Emmerik & Kamphuis, 2011) although one study of accident survivors found a lower rate of PTSD under ICD-11 (O'Donnell et al., 2014). All studies so far have found some evidence of reduced comorbidity with depression under ICD-11.

These developments have given greater urgency to the need to understand core symptoms such as flashbacks and made them a prime target for research. From a cognitive perspective the vividness and immediacy of the symptom is interesting as these features are likely to influence a wide variety of memory judgments, for example, concerning the veracity of the intrusive images experienced (Brewin et al., 2012). In addition, their considerable clinical impact suggests that a focus on this symptom has the potential to bring about substantial gains in therapeutic efficacy.

## Supplementary Material

Re-experiencing traumatic events in PTSD: new avenues in research on intrusive memories and flashbacks

Re-experiencing traumatic events in PTSD: new avenues in research on intrusive memories and flashbacks

Re-experiencing traumatic events in PTSD: new avenues in research on intrusive memories and flashbacks

Re-experiencing traumatic events in PTSD: new avenues in research on intrusive memories and flashbacks

Re-experiencing traumatic events in PTSD: new avenues in research on intrusive memories and flashbacks

Re-experiencing traumatic events in PTSD: new avenues in research on intrusive memories and flashbacks
