# Supplementary material for: Re-experiencing traumatic events in PTSD: new avenues in research on intrusive memories and flashbacks
Source: Eur J Psychotraumatol. 2015 May 19;6:10.3402/ejpt.v6.27180. doi: 10.3402/ejpt.v6.27180 (PMC4439411; doi:10.3402/ejpt.v6.27180)
Supplement: Re-experiencing traumatic events in PTSD: new avenues in research on intrusive memories and flashbacks [file EJPT-6-27180-s001.pdf]

Revivre l’évènement traumatique avec un ESPT: Nouvelles avancées dans la recherche sur les mémoires intrusives et les flashbacks.

Chris R. Brewin

Les flashbacks post-traumatiques, qui consistent en une ré-expérience intrusive d’un vécu traumatique dans le présent, ont été mieux définis pour la première fois par le DSM-5 et ont été identifiés comme étant un symptôme unique de l’ESPT dans les critères diagnostic de l’ICD-11. Cependant peu de recherches portant sur les flashbacks ont été conduites et de nouveaux efforts sont nécessaires pour comprendre les bases cognitives et biologiques de cet important symptôme. Il existe de plus un intérêt particulier pour la recherche sur l’évaluation des flashbacks, et sur la manière dont ces flashbacks surviennent dans différents contextes comme les psychoses ou soins intensifs.

Mots-clés: état de stress post-traumatique; mémoire; flashbacks

Citation: European Journal of Psychotraumatology 2015, **6**: 27180 - http://dx.doi.org/10.3402/ejpt.v6.27180
