# Supplementary material for: Re-experiencing traumatic events in PTSD: new avenues in research on intrusive memories and flashbacks
Source: Eur J Psychotraumatol. 2015 May 19;6:10.3402/ejpt.v6.27180. doi: 10.3402/ejpt.v6.27180 (PMC4439411; doi:10.3402/ejpt.v6.27180)
Supplement: Re-experiencing traumatic events in PTSD: new avenues in research on intrusive memories and flashbacks [file EJPT-6-27180-s002.pdf]

Wiederleben traumatischer Ereignisse in der PTBS: neue Wege in der Erforschung intrusiver Erinnerungen und flashbacks

Chris.R.Brewin

Posttraumatische flashbacks, die aus intrusiven Wiedererleben der traumatischen Erfahrungen in der Gegenwart bestehen, sind erstmals im DSM-5 klarer definiert worden und wurden im ICD-11 Vorschlag als einzigartiges Symptom der PTBS identifiziert. Dennoch gibt es bislang relativ wenig Forschung zu den flashbacks, neue Forschungsanstrengungen sind daher notwendig, um die kognitive und biologische Basis dieses wesentlichen Symptoms zu verstehen. Darüberhinaus gibt es einen beachtlichen Forschungsbereich hinsichtlich der Diagnostik von flashbacks und deren Auftreten in unterschiedlichen Kontexten z. B. als Psychose oder in der Intensivpflege.

Schlüsselwörter: Posttraumatische Belastungsstörungen, Erinnerungen, flashbacks

Citation: European Journal of Psychotraumatology 2015, **6**: 27180 - http://dx.doi.org/10.3402/ejpt.v6.27180
