# Supplementary material for: Re-experiencing traumatic events in PTSD: new avenues in research on intrusive memories and flashbacks
Source: Eur J Psychotraumatol. 2015 May 19;6:10.3402/ejpt.v6.27180. doi: 10.3402/ejpt.v6.27180 (PMC4439411; doi:10.3402/ejpt.v6.27180)
Supplement: Re-experiencing traumatic events in PTSD: new avenues in research on intrusive memories and flashbacks [file EJPT-6-27180-s003.pdf]

A traumatikus élmény újraélése PTSD-ben: Új kutatási témák az intruzív emlékek és flashbackek kapcsán

Chris R. Brewin

A poszttraumás flashbackek, amelyek a traumás élmények jelenben való intruzív újraélését jelentik, most először pontosabb meghatározást kaptak a DSM-5-ben, illetve mint a PTSD-re kifejezetten jellemző tünetként kerül bevezetésre majd az BNO-11-ben. Aránylag kevés kutatást végeztek a flashbackek jobb megértésére, ugyanakkor fontos lenne új vizsgálatokban igazolni a jelenség kognitív és biológiai hátterét. Továbbá, tere lenne annak, hogy empirikusan igazolt vizsgáló módszerek legyenek a flashbackek felmérésére, valamint a flashbackek különböző kontextusban való megjelenésének kutatására (pl. pszichózis vagy intenzív ellátás).

Kulcsszavak: poszttraumás stressz zavar; emlékezet; flashback

Citation: European Journal of Psychotraumatology 2015, **6**: 27180 - http://dx.doi.org/10.3402/ejpt.v6.27180
