# Supplementary material for: Re-experiencing traumatic events in PTSD: new avenues in research on intrusive memories and flashbacks
Source: Eur J Psychotraumatol. 2015 May 19;6:10.3402/ejpt.v6.27180. doi: 10.3402/ejpt.v6.27180 (PMC4439411; doi:10.3402/ejpt.v6.27180)
Supplement: Re-experiencing traumatic events in PTSD: new avenues in research on intrusive memories and flashbacks [file EJPT-6-27180-s004.pdf]

Re-experimentando eventos traumáticos en el TEPT: Nuevas vías en la investigación sobre recuerdos intrusivos y *flashbacks*

Chris R. Brewin

Los *flashbacks* postraumáticos, que consisten en la reexperimentación intrusiva de experiencias traumáticas en el presente, se han definido claramente por primera vez en el DSM-5 y se han identificado como un síntoma singular del TEPT en los criterios diagnósticos propuestos en la CIE-11. Sin embargo, se ha llevado a cabo relativamente poca investigación sobre *flashbacks* y se requieren nuevos esfuerzos de investigación para comprender las bases cognitivas y biológicas de este importante síntoma. Además hay un considerable campo de estudio sobre cómo se deben evaluar los *flashbacks* y sobre los *flashbacks* que ocurren en diferentes contextos, como psicosis o cuidados intensivos.

Palabras clave: Trastorno de estrés postraumático; memoria; flashbacks

Citation: European Journal of Psychotraumatology 2015, **6**: 27180 - **http://dx.doi.org/10.3402/ejpt.v6.27180**
