# Supplementary material for: Re-experiencing traumatic events in PTSD: new avenues in research on intrusive memories and flashbacks
Source: Eur J Psychotraumatol. 2015 May 19;6:10.3402/ejpt.v6.27180. doi: 10.3402/ejpt.v6.27180 (PMC4439411; doi:10.3402/ejpt.v6.27180)
Supplement: Re-experiencing traumatic events in PTSD: new avenues in research on intrusive memories and flashbacks [file EJPT-6-27180-s005.pdf]

TSSB'de Travmatik Olayları Yeniden Deneyimleme: Dalıcı Anılar ve Geri dönüşler üzerine Araştırmalarda Yeni Yollar

Chris R. Brewin

Travma sonrası geri dönüşler, şimdiki anda travmatik olayı dalıcı şekilde tekrar yaşamaları da içeren, DSM-5'te ilk kez açık şekilde tanımlanmış ve ICD-11 tanı kriterlerine göre TSSB'nin tek belirtisi olarak tanımlanmıştır. Göreceli olarak geri dönüşler üzerine çok az çalışma yürütülmüş olup ve bu önemli belirtinin bilişsel ve biyolojik temellerini anlamak için yeni araştırma çabaları gerekmektedir. Bunun yanında, geri dönüşlerin nasıl değerlendirilmesi gerektiği konusunda ve psikoz ve yoğun bakım gibi farklı bağlamlarda oluşan geri dönüşler ile ilgili oldukça önemli araştırma alanları mevcuttur.

Anahtar Kelimeler: Travma sonrası stres bozukluğu; hafıza; geri dönüşler

Citation: European Journal of Psychotraumatology 2015, **6**: 27180 - http://dx.doi.org/10.3402/ejpt.v6.27180
