# Supplementary material for: Re-experiencing traumatic events in PTSD: new avenues in research on intrusive memories and flashbacks
Source: Eur J Psychotraumatol. 2015 May 19;6:10.3402/ejpt.v6.27180. doi: 10.3402/ejpt.v6.27180 (PMC4439411; doi:10.3402/ejpt.v6.27180)
Supplement: Re-experiencing traumatic events in PTSD: new avenues in research on intrusive memories and flashbacks [file EJPT-6-27180-s006.pdf]

Ponovno proživljavanje traumatksih događaja u PTSP-u: novi pravci u istraživanju sećanja koje se nameću i flešbek-ova

Chris R. Brewin

Posttraumatski flešbek-ovi, koji se sastoje od intruzivnog ponovnog proživljavanja traumatskog iskustva u sadašnjosti, prvi put su jasnije definisani u DSM-5 klasifikaciji i prepoznati su kao jedinstven simptom PTSP-a u predloženim MKB-10 dijagnostičkim kriterijumima. Sprovedeno je relativno malo istraživanja o flešbek-ovima i potrebni su novi istraživački poduhvati da bi se razumele kognitivne i biloške osnove ovog važnog simptoma. Kao dodatak, postoji značajan istraživački prostor za razumevanje kako bi flešbek-ove trebalo procenjivati, kao i flešbek-ove koji se pojavljuju u različitim kontekstima, kao što su psihoze ili odeljenja intenzivne nege.

Ključne reči: posttraumatski stresni poremećaj; pamćenje; flešbekovi

Citation: European Journal of Psychotraumatology 2015, **6**: 27180 - http://dx.doi.org/10.3402/ejpt.v6.27180
